# Influences of Heider Balance on Knowledge, Attitude, Practice, and Quality of Life in Bladder Cancer Patients after Urinary Diversion

**DOI:** 10.1155/2022/5635971

**Published:** 2022-12-13

**Authors:** Hao-yu Zou, Liu-yi Zhang, Yue-lan Qin, Ping Li, Li Zhang, Ke Yang

**Affiliations:** ^1^Department of Nursing, Hunan Provincial People's Hospital, The First Affiliated Hospital of Hunan Normal University, Changsha, China; ^2^Department of Nursing, Medical College of Hunan Normal University, Changsha, China; ^3^Department of Urology, Hunan Provincial People's Hospital, The First Affiliated Hospital of Hunan Normal University, Changsha, China

## Abstract

**Objective:**

To explore the influences of Heider balance on knowledge, attitude, practice (KAP), and quality of life in bladder cancer patients after urinary diversion.

**Methods:**

A set of bladder cancer patients after urinary diversion in our hospital from January 2016 to December 2020 were included in this study. Patients who received out-hospital intervention based on Heider balance were included in the observation group (85 cases). Meanwhile, patients who received routine out-hospital intervention were included in the control group (85 cases), and these patients matched with the observation group by gender, age, and education level. The scores of KAP, WHO quality of life-100 (WHOQOL-100) before discharge and at 6 months after discharge, and the rate of complications were compared in the two groups.

**Results:**

At 6 months after discharge, the score of these items of KAP including basic knowledge of disease, procedure of pouch replacement, dealing with pouch leakage, skin care of stoma, purchase and storage of pouch, dealing with stoma complications, optimistic mentality for disease, optimistic mentality for stoma, trust in medical staff, willingness to correct bad habits, confidence in maintaining health behavior, maintaining in health dietary habit, maintaining in health behavior, learning from relevant books, learning from relevant videos, experienced in pouch replacement, and experienced in care of stoma of the observation group were significantly higher than those of the control group (*t* = 6.144, 9.366, 3.129, 3.809, 4.173, 5.923, 2.788, 8.871, 3.291, 10.797, 7.067, 7.805, 3.828, 9.454, 2.827, 4.059, and 8.662, respectively, all *P* < 0.05). The scores of 16 items of WHOQOL-100 such as energy and fatigue, sleep and rest, positive feelings, thinking, learning, memory and concentration, self-esteem, body image and appearance, negative feelings, mobility, activities of daily living, dependence on medical support, personal relationships, social support, health and social care: availability and quality, opportunities to get new information/skills, opportunities for recreation and leisure, and quality of life from viewpoint in the observation group were significantly higher than those in the control group (*t* = 2.666, 2.571, 2.961, 3.453, 4.279, 2.781, 3.775, 4.807, 5.850, 4.194, 3.324, 3.873, 5.118, 3.244, 2.956, and 4.218, respectively, all *P* < 0.05). The rate of complications of the observation group was significantly lower than that of the control group (*x*^2^ = 5.829, *P* < 0.05).

**Conclusion:**

The Heider balance can help to reduce the rate of complications, improve knowledge, attitude, practice, and quality of life in urinary diversion patients. These merits make it an attractive approach in guidance of out-hospital intervention.

## 1. Introduction

Bladder cancer is one of the most common types of urinary cancer, the urinary diversion is routinely performed after radical cystectomy for the muscle-invasive or high-risk metastatic bladder cancer [1]. After urinary diversion, the urostomy is a stoma created to divert urine in the abdominal wall after the bladder has been resected. Subsequently, the functional and physical changes of cancer patients will result in psychological anxiety and sense of inferiority, which may threaten their mental and physical health [2, 3]. There is an agreement that the knowledge, attitude, and practice (KAP) of patients play a vital role in the management of chronic diseases after discharge, and some studies concluded that the self-care ability and quality of life will be improved by mean of the strengthening of patients' KAP [4, 5]. However, the age of bladder cancer patients was relatively older, with the problems such as poor acceptance, rigid thinking, and inflexible behavior styles [6], which made it difficult for them to achieve satisfying adaptation in psychology and behavior.

The Heider balance was also well-known as the “P-O-X” model, which was originally proposed by Heider in 1946 [7]. The concept of Heider balance mainly involved an equilibrium analysis in terms of positive (friendly or agreement) and negative (hostile or disagreement) relations in a triad, and it was correlated with each other by the connection of the three-body of “P-O-X” [8]. The Heider balance constructed a novel perspective on the epistemological relation among subject, object, and medium, and created a new conceptual foundation for psychological and behavioral intervention [9]. In the past decades, the conventional out-hospital intervention usually focused on the patients and ignored the attention to their caregiver's influences. In this study, the patients, their caregivers, and urostomy care were respectively supposed to be the subject (P), object (O), and medium (X). Then, the out-hospital intervention based on Heider balance was performed to preliminarily investigate its influences on KAP, quality of life, and complications in bladder cancer patients after urinary diversion.

## 2. Participants and Methods

### 2.1. Participants

A series of bladder cancer patients who received urinary diversion in our hospital from January 2016 to December 2020 were enrolled in this study, and this study was approved by the Medical Ethics Committee of Hunan Provincial People's Hospital (No: 2016-002). A prespecified calculation was performed to test the sample size. The method of calculation was similar to the previous study [10], with the *α* set at 0.05 (two-tailed test) and the power set at 0.9 (1 − *β*), and *σ* represents the estimated value of the standard deviation of quality of life between the two groups. It showed that the target sample size should be 164 cases (82 cases in each group). With an estimation to a total of 20% drop out and attrition rate, a sample size with ≥198 cases should be included. Inclusion criteria in this study were set below: (1) patients with bladder cancer who underwent radical resection and urinary diversion; (2) patients without communication disorders and cognitive impairments; and (3) patients who volunteered to participate in this study. Exclusion criteria were listed as below: (1) patients who dropped out during the period of observation; (2) patients with other severe comorbidities; and (3) patients with a life expectancy of less than 12 months. According to the above criteria, 206 eligible patients were initially enrolled in this study. Of these patients, 101 cases received the out-hospital intervention based on Heider balance were assigned to the observation group, and another 105 cases received routine out-hospital intervention were assigned to the control group. At the end of observation, 85 cases were ultimately included and 16 cases were excluded due to loss to follow-up (8 cases), refusal to cooperate (5 cases), and died of worsening disease (3 cases), respectively. Meanwhile, another 85 patients matched in 1 : 1 ratio with the observation group by gender, age (±5 years), and education level, and 20 cases were excluded due to loss to follow-up (7 cases), refusal to cooperate (6 cases), died of worsening disease (4 cases), unmatched with gender or education level (3 cases), respectively. Finally, 170 cases were included in this study (85 cases in each group).

### 2.2. Methods

**Figure 1:**
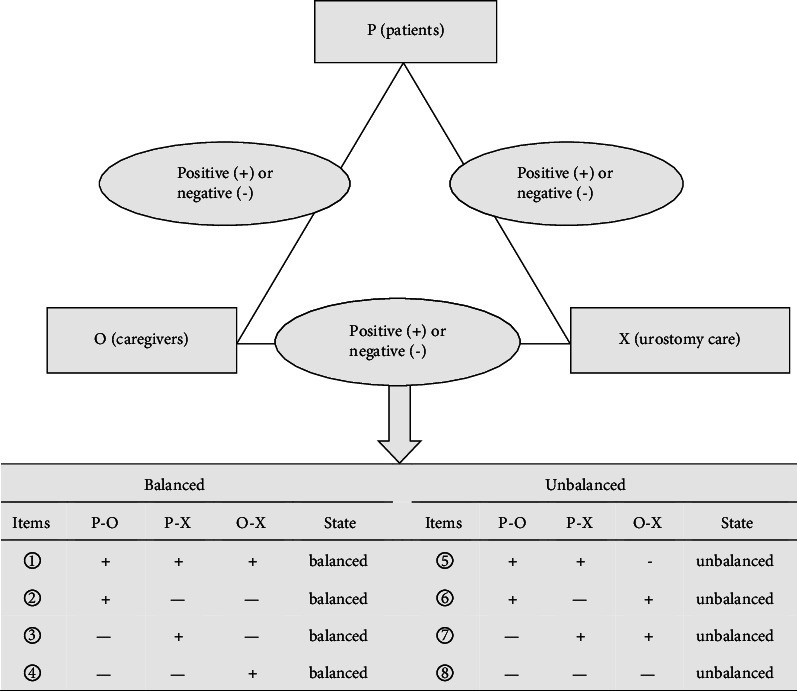
The structure of Heider balance and assessment of status.

All patients received radical cystectomy and urinary diversion, routine treatment nursing, and related health education. Before discharge, all patients were asked to learn the pouch replacement, stoma nursing, precautions in dietary and bathing, daily activities, and other related knowledge and skills through demonstration or session. According to the framework of Heider balance [11], “the patients, caregivers, urostomy care” were set as “P, O, X,” and a triangular unit of Heider balance with patient (P), caregiver (O), and urostomy care (X) was established. Thus, a mutual correlation among “P, O, X” such as “a friend of my friend is my friend, an enemy of my friend is my enemy” was produced in “P-O, O-X, P-X.” A “+” for a positive relationship (friendly or like) and a “−” for a negative relationship (hostile or dislike) were assigned to the reciprocal relationship between the two elements in a triad unit. If the product of the three edges is “+1,” indicating that the triad unit is balanced. Otherwise, the product is “−1,” indicating that the triad unit is unbalanced. The “+, +, +” of the three edges denoted everybody has a positive effect on each other, which was considered as the best status of “P-O-X” [12]. As shown in Figure 1.

After the establishment of “P-O-X,” the status of “P-O-X” was assessed through the relationships among patients, caregivers, and urostomy care. If the relationship is not in a balanced status, the reasons of the unbalanced status should be detected, and subsequent rectifying measures need to be carried out. For instance, a caregiver lacked nutrition knowledge and cooking skills, he/she was tired of preparing proper dietary for him/her patient, and the relationship of “P-O” was assigned as “−.” Then, a health education program focused on nutrition knowledge and cooking skills should be performed for this caregiver. After discharge, all patients were followed-up for six months at least, with the telephone follow-up or return visit once a week in the first month, and once a month in subsequent months. During the out-hospital intervention, the status of patients, caregivers, and urostomy care was evaluated per month at least, and the unbalanced status was rectified under the supervision of responsible staff.

### 2.3. Study Parameters

Baseline data (age, gender, education status, family income, and others) were collected at the time of entry into this study, and the scores of questionnaires were collected under the guidance of medical staff. A 30-item questionnaire was used to evaluate the KAP of urostomy patients, which includes 12 items of stoma knowledge (including basic knowledge of disease, basic knowledge of stoma operation, observation of stoma, and others), 9 items of health belief (including optimistic mentality for disease, optimistic mentality for stoma, enthusiasm for rehabilitation plan, and others), 9 items of health behavior (including maintaining in health dietary habit, maintaining in health behavior, learning from relevant books, and others) [13], and each item was scored by a Likert scale ranging from point 1 (very poor) to 5 (very good). The WHO Quality of Life-100 (WHOQOL-100) was used to evaluate quality of life of patients, which includes six domains of quality of life: physical, psychological, level of independence, social relation, environment, and spirituality [14]. These six domains contain 24 facets, each facet includes 4 items, and each item was also scored by a Likert scale ranging from point 1 to 5, with the higher scores indicating the better quality of life.

### 2.4. Statistical Analysis

The data were processed by SPSS25.0 software. The measurement data were presented as mean ± standard deviation ($\bar{x}\pm s$), by using *t*-test. The counting data were expressed as frequency and percentages (%) by using *x*^2^ test. The ranked data were analyzed by the rank sum test. *P* < 0.05 indicated that the difference was significant.

## 3. Results

### 3.1. Baseline Data of Patients

There were no significant differences in gender, age, education status, marital status, and urostomy type between the two groups (*P* > 0.05). As shown in Table 1.

### 3.2. Comparisons of KAP

Before discharge, there were no statistically significant differences in the score of each item of KAP between the two groups (*P* > 0.05). At 6 months after discharge, the score of 6 items (including basic knowledge of disease, procedure of pouch replacement, dealing with pouch leakage, skin care of stoma, purchase and storage of pouch, and dealing with stoma complications) in knowledge, 5 items (including optimistic mentality for disease, optimistic mentality for stoma, trust in medical staff, willingness to correct bad habits, confidence in maintaining health behavior) in attitude, and 6 items (including maintaining in health dietary habit, maintaining in health behavior, learning from relevant books, learning from relevant videos, experienced in pouch replacement, experienced in care of stoma) in behavior of the observation group were significantly higher than those of the control group, these differences were statistically significant (*P* < 0.05). As shown in Table 2.

### 3.3. Comparisons of Quality of Life

Before discharge, no significant differences were observed in the WHOQOL-100 score between the two groups (*P* > 0.05). At 6 months after discharge, the energy and fatigue, sleep and rest, positive feelings, thinking, learning, memory and concentration, self-esteem, body image and appearance, negative feelings, mobility, activities of daily living, dependence on medical support, personal relationships, social support, health and social care: availability and quality, opportunities to get new information/skills, opportunities for recreation and leisure, and quality of life from viewpoint in the observation group were higher than those in the control group, the differences were statistically significant (*P* < 0.05). As shown in Table 3.

### 3.4. Comparisons of Complications

During the course of observation, 14 cases of stoma complications occurred in the observation group, including 5, 3, 2, 2, 1, and 1 cases with dermatitis, stoma infection, skin stripping, stoma retraction, stoma stenosis, and fistula, respectively. Among them, 5 cases occurred with 3 complications, involving 9 patients, accounting for 10.59% (9/85). In the control group, 27 cases of complications occurred, including 9, 5, 4, 3, 3, 2, and 1 cases with dermatitis, stoma infection, skin stripping, stoma stenosis, stoma retraction, granulomatosis, and fistula, respectively. Among them, 6 cases occurred with 2 complications, involving 21 patients, accounting for 24.71% (21/85). The rate of complications in the observation group was lower than that in the control group, and the difference was statistically significant (*x*^2^ = 5.829, *P*=0.016).

## 4. Discussion

The KAP is a way to inspect the changes in people's thinking and behavior. Evidence from a study showed that knowledge was the foundation of modifications in attitude and behavior, and a good attitude would further encourage positive modifications in behavior [15]. The application of Heider balance was an assumption thinking that any edge of triads was equivalently important in the dynamics of network among patients, disease, and caregivers, and patients can obtain good care support from their caregivers [16]. Just because urostomy care always has a long-lasting burden for patients and their caregivers, patients are easy to suffer from inadequate care support from medical institutions after discharge, and almost half of patients sought the help of professional urostomy care, especially in these patients with permanent urostomy [17].

In this study, relationships among a focal person (patients, P), another person (caregivers, O), and a medium (urostomy care, X) are the main focus of Heider balance. There are eight possible component statuses, including four balanced and four unbalanced, and a relationship being positive or negative depends on the cognitive perception of each other [18]. Among eight statuses, the “P + O, P + X, O + X” is considered as the best status of “P-O-X” model with positive influence on each other. For example, the triad unit was in an unbalanced status with “P − O, P + X, O + X,” and the reason of “P − O” was that the caregiver (O) had a disgusting attitude for the uncomfortable features of pouch drainage from patients (P). Particularly, more attention should be paid to “P − O,” and a health education program or kind conversation would be performed to rectify the negative attitude between caregivers and patients. According to the mechanism mentioned above, we can draw a similar conclusion that any other unbalanced status can be turned by reversing anyone's attitude in this triad unit, which may be responsible for the feasibility of out-hospital intervention based on this model [19].

After six months of out-hospital intervention, the scores of 6 items in knowledge, 5 items in attitude, and 6 items in behavior of the observation group were significantly higher than those of the control group, which suggested that the out-hospital intervention based on Heider balance can obviously modify the knowledge, attitude, and practice comparing with the routine out-hospital intervention. A qualitative systematic review considered that good family support had a positive impact on patients, which can promote positive outcomes in many chronic diseases [20]. Perhaps, this viewpoint was in accordance with the principle of Heider balance. Furthermore, in a study on Heider balance performed by Lou and Shang [21], they also held the viewpoint that the caregivers can improve knowledge and attitude towards pain management in cancer patients, and followingly increase the efficacy of out-hospital intervention, which were similar to the results in our study.

In our study, the findings demonstrated that most of items of WHOQOL-100 in the observation group were significantly higher than those of the control group, especially in physical health, psychological, independence level, and social relations domain. These results indicated that the out-hospital intervention based on Heider balance can improve quality of life of patients. As others reported, the education program not only improves patients' quality of life but also makes some promotions in self-management after urostomy [22], and the stoma education and out-hospital intervention teams should be early created and maintained for six months [23]. Furthermore, a randomized controlled trial performed by Jensen et al. [24] found that a short-term preoperative stoma intervention can facilitate rehabilitation in stoma patients. In this study, the early intervention began with decision-making for operation and maintained for six months. Our results showed that the scores of KAP and quality of life, complications in the observation group were superior to the control group. Nevertheless, no significant differences were observed in the environment domain except “health and social care: availability and quality, opportunities to get new information/skills, and opportunities for recreation and leisure.” The possible explanation may be that some environmental factors such as home environment, financial resources, and transportation are hard to rectify within a short time.

In recent years, study regarding the out-hospital intervention of quality of life still is a hot topic in patients with urostomy, and many researchers have performed a lot of work on it. A study indicated that postoperative home community follow-up by teleconsultation will decrease their burden and the readmission rate of stoma patients [25]. Another study considered that the care team intervention can reduce complications and improve self-efficacy level in persons with urostomy [26]. As Stavropoulou et al. pointed out [27], these improvements may be correlated with promotions in family and organizational support, self-management and empowerment, and patients' autonomy. Although our findings were supported by the above studies in some way, some shortcomings should be presented here: some potential statistical biases cannot be completely avoided owing to the small sample, so the results need to be verified in the multicentre study with large samples. Furthermore, this study was a preliminary exploration of the out-hospital intervention based on Heider balance, and the standard protocol of clinical pathway for out-hospital intervention based on Heider balance should be supplemented in future study.

In summary, the out-hospital intervention based on Heider balance can help to reduce the rate of complications, improve patients' knowledge, attitude, and practice, and promote quality of life in urinary diversion patients. These merits make the Heider balance an attractive approach in the guidance of out-hospital intervention.

## Figures and Tables

**Table 1:** Comparisons of baseline data between the two groups [*n* (%)].

Items	Observation group (*n* = 85)	Control group (*n* = 85)	*χ* ^2^ */Z*	*P*
Gender			—	1.000
Male	63 (74.12)	63 (74.12)		
Female	22 (25.88)	22 (25.88)		
Age (years)			1.071	0.284
<44	8 (9.41)	12 (14.12)		
44–59	32 (37.65)	34 (40)		
≥60	45 (52.94)	39 (45.88)		
Education level			—	1.000
Elementary	16 (18.82)	16 (18.82)		
Junior/senior high	44 (51.76)	44 (51.76)		
College/university	25 (29.41)	25 (29.41)		
Marital status			1.978	0.372
Married	55 (64.71)	46 (54.12)		
Divorced	16 (18.82)	21 (24.71)		
Widowed	14 (16.47)	18 (21.18)		
Family income (CNY/month)			0.030	0.976
<10000	16 (18.82)	14 (16.47)		
10000–20000	46 (54.12)	50 (58.82)		
>20000	19 (22.35)	16 (18.82)		
Not informed	4 (4.71)	5 (5.88)		
Caregivers			2.177	0.140
Family members	62 (72.94)	53 (62.35)		
Employed caregivers	23 (27.06)	32 (37.65)		
Comorbidities			0.096	0.953
Hypertension	38 (44.71)	36 (42.35)		
Diabetes	25 (29.41)	26 (30.59)		
Others^*∗*^	22 (25.88)	23 (27.06)		
Urostomy type			0.956	0.620
Ileal conduit	76 (89.41)	74 (87.06)		
Bilateral ureterostomy	4 (4.71)	7 (8.24)		
Unilateral ureterostomy	5 (5.88)	4 (4.71)		

Note: ^*∗*^Including common gout, gastrointestinal diseases, pulmonary diseases, and others.

**Table 2:** Comparisons of scores of KAP between the two groups [n(%)].

Items	Before discharge				At 6 months after discharge			
	Observation group (*n* = 85)	Control group (*n* = 85)	*t*	*P*	Observation group (*n* = 85)	Control group (*n* = 85)	*t*	*P*
Knowledge								
Basic knowledge of disease	2.21 ± 0.74	2.19 ± 0.78	0.202	0.840	4.32 ± 0.62	3.67 ± 0.75	6.144	<0.001
Basic knowledge of stoma operation	3.07 ± 0.83	3.18 ± 0.80	0.846	0.399	4.05 ± 0.72	4.12 ± 0.75	0.627	0.532
Observation of stoma	2.51 ± 0.70	2.39 ± 0.64	1.145	0.254	3.98 ± 0.58	3.87 ± 0.61	1.160	0.248
Procedure of pouch replacement	3.38 ± 0.72	3.59 ± 0.85	1.750	0.082	4.46 ± 0.55	3.59 ± 0.66	9.366	<0.001
Emptying of pouch drainage	3.02 ± 0.77	2.87 ± 0.69	1.366	0.174	4.52 ± 0.50	4.40 ± 0.52	1.505	0.134
Measuring of stoma size	3.68 ± 0.80	3.74 ± 0.80	0.477	0.634	4.47 ± 0.55	4.36 ± 0.53	1.280	0.202
Connecting and shutting of pouch	2.60 ± 0.89	2.59 ± 0.79	0.091	0.927	4.15 ± 0.72	4.02 ± 0.64	1.246	0.214
Application of ancillary devices	2.14 ± 0.58	2.20 ± 0.59	0.653	0.515	4.02 ± 0.58	3.92 ± 0.54	1.236	0.218
Dealing with pouch leakage	1.98 ± 0.56	2.02 ± 0.62	0.523	0.602	3.82 ± 0.73	3.45 ± 0.84	3.129	0.002
Skin care of stoma	2.66 ± 0.66	2.58 ± 0.66	0.810	0.419	4.32 ± 0.58	3.96 ± 0.63	3.809	<0.001
Purchase and storage of pouch	2.76 ± 0.73	2.85 ± 0.72	0.741	0.460	4.16 ± 0.57	3.75 ± 0.71	4.173	<0.001
Dealing with stoma complications	1.88 ± 0.52	1.84 ± 0.57	0.559	0.577	3.72 ± 0.84	2.95 ± 0.84	5.923	<0.001
Attitude								
Optimistic mentality for disease	2.41 ± 0.66	2.47 ± 0.59	0.613	0.541	4.04 ± 0.71	3.69 ± 0.87	2.788	0.006
Optimistic mentality for stoma	2.71 ± 0.72	2.68 ± 0.71	0.214	0.831	3.92 ± 0.71	2.98 ± 0.67	8.871	<0.001
Enthusiasm for rehabilitation plan	3.12 ± 0.86	3.02 ± 0.84	0.718	0.474	3.53 ± 0.72	3.36 ± 0.72	1.493	0.137
Trust in medical staff	3.61 ± 0.82	3.64 ± 0.83	0.186	0.852	4.27 ± 0.56	3.96 ± 0.64	3.291	0.001
Trust in their caregivers	3.26 ± 0.77	3.31 ± 0.8	0.389	0.698	4.26 ± 0.64	4.12 ± 0.64	1.435	0.153
Willingness to correct bad habits	3.18 ± 0.64	3.05 ± 0.63	1.324	0.187	4.45 ± 0.52	3.51 ± 0.61	10.797	<0.001
Willingness to help others	2.84 ± 0.75	2.87 ± 0.75	0.306	0.760	2.94 ± 0.7	3.06 ± 0.68	1.116	0.266
Willingness to correct negative emotion	2.09 ± 0.61	2.12 ± 0.57	0.261	0.794	4.16 ± 0.69	4.04 ± 0.73	1.189	0.236
Confidence in maintaining health behavior	2.87 ± 0.65	2.81 ± 0.66	0.583	0.560	4.42 ± 0.54	3.76 ± 0.67	7.067	<0.001
Behavior								
Maintaining in health dietary habit	2.42 ± 0.56	2.53 ± 0.61	1.176	0.241	4.33 ± 0.52	3.64 ± 0.63	7.805	<0.001
Maintaining in health behavior	2.59 ± 0.64	2.64 ± 0.67	0.468	0.641	4.19 ± 0.57	3.84 ± 0.63	3.828	<0.001
Learning from relevant books	1.78 ± 0.45	1.75 ± 0.46	0.338	0.736	3.35 ± 0.57	2.58 ± 0.50	9.454	<0.001
Learning from relevant videos	1.85 ± 0.48	1.91 ± 0.48	0.804	0.423	3.57 ± 0.92	3.19 ± 0.83	2.827	0.005
Experienced in pouch replacement	2.40 ± 0.73	2.46 ± 0.80	0.498	0.619	4.07 ± 0.65	3.60 ± 0.85	4.059	<0.001
Experienced in care of stoma	2.08 ± 0.56	2.11 ± 0.58	0.269	0.788	4.45 ± 0.52	3.76 ± 0.50	8.662	<0.001
Communicated with other patients	1.96 ± 0.64	2.08 ± 0.66	1.177	0.241	3.07 ± 0.83	3.16 ± 0.90	0.711	0.478
Complying with doctor's advice	3.58 ± 0.88	3.54 ± 0.89	0.260	0.795	3.82 ± 0.80	3.73 ± 0.85	0.741	0.460
Return visit	—	—	—	—	4.35 ± 0.61	4.21 ± 0.60	1.520	0.130

**Table 3:** Comparisons of quality of life between the two groups ($\bar{x}\pm s$).

Domain	Before discharge				At 6 months after discharge			
	Observation group (*n* = 85)	Control group (*n* = 85)	*t*	*P*	Observation group (*n* = 85)	Control group (*n* = 85)	*t*	*P*
Physical health								
Pain and discomfort	13.99 ± 3.04	13.72 ± 3.20	0.565	0.573	11.55 ± 2.88	12.22 ± 2.72	1.561	0.120
Energy and fatigue	13.12 ± 2.95	13.28 ± 2.15	0.415	0.678	15.26 ± 2.84	14.25 ± 2.04	2.666	0.008
Sleep and rest	12.92 ± 3.12	12.13 ± 3.26	1.611	0.109	14.94 ± 2.05	13.95 ± 2.89	2.571	0.011
Psychological								
Positive feelings	10.13 ± 2.83	9.84 ± 2.44	0.725	0.469	14.41 ± 3.09	13.00 ± 3.12	2.961	0.004
Thinking, learning, memory and concentration	13.56 ± 3.14	13.24 ± 3.32	0.665	0.507	14.61 ± 2.41	13.11 ± 3.22	3.453	0.001
Self-esteem	12.32 ± 3.25	12.56 ± 3.01	0.514	0.608	14.15 ± 2.00	12.71 ± 2.39	4.279	<0.001
Body image and appearance	9.11 ± 1.51	8.73 ± 1.56	1.597	0.112	13.85 ± 3.28	12.35 ± 3.72	2.781	0.006
Negative feelings	13.80 ± 3.31	13.87 ± 2.99	0.146	0.884	11.79 ± 2.42	13.31 ± 2.81	3.775	<0.001
Independence level								
Mobility	10.81 ± 2.78	10.78 ± 2.11	0.093	0.926	13.89 ± 2.83	12.01 ± 2.24	4.807	<0.001
Activities of daily living	11.84 ± 2.53	12.36 ± 2.79	1.295	0.197	15.00 ± 3.20	12.38 ± 2.62	5.850	<0.001
Dependence on medical support	15.24 ± 2.92	14.48 ± 3.11	1.626	0.106	11.66 ± 2.78	13.60 ± 3.24	4.194	<0.001
Work capacity	12.09 ± 2.65	12.41 ± 2.81	0.758	0.450	11.75 ± 3.04	12.36 ± 3.37	1.243	0.216
Social relations								
Personal relationships	13.91 ± 2.84	13.76 ± 3.12	0.309	0.758	16.52 ± 2.10	15.36 ± 2.41	3.324	0.001
Social support	13.55 ± 3.15	13.75 ± 3.31	0.404	0.687	16.78 ± 2.36	15.14 ± 3.09	3.873	<0.001
Sexual activity	12.87 ± 2.96	13.38 ± 2.69	1.165	0.246	9.01 ± 2.40	9.52 ± 2.76	1.274	0.205
Environment								
Physical safety and security	12.81 ± 2.64	13.04 ± 2.47	0.570	0.570	12.05 ± 3.33	11.74 ± 3.39	0.593	0.554
Home environment	12.34 ± 2.83	12.28 ± 2.82	0.136	0.892	12.28 ± 2.80	12.80 ± 2.95	1.174	0.242
Financial resources	13.71 ± 3.14	13.07 ± 3.17	1.313	0.191	13.61 ± 2.95	12.94 ± 3.17	1.429	0.155
Health and social care: availability and quality	11.99 ± 3.50	12.12 ± 3.19	0.252	0.801	14.08 ± 2.80	11.73 ± 3.18	5.118	<0.001
Opportunities to get new information/skills	9.93 ± 2.59	10.35 ± 2.97	0.989	0.324	10.93 ± 3.07	12.54 ± 3.40	3.244	0.001
Opportunities for recreation and leisure	11.60 ± 3.29	12.47 ± 3.62	1.640	0.103	12.36 ± 3.00	13.86 ± 3.57	2.956	0.004
Physical environment	10.87 ± 3.17	11.38 ± 3.00	1.068	0.287	12.45 ± 3.39	12.09 ± 3.04	0.714	0.476
Transportation	10.05 ± 3.02	9.93 ± 2.69	0.269	0.789	10.12 ± 2.89	10.36 ± 3.63	0.491	0.624
Spiritual aspects/spirituality/religion/personal beliefs	15.48 ± 2.75	15.54 ± 3.22	0.128	0.898	15.61 ± 2.79	15.99 ± 2.64	0.903	0.368
Quality of life from viewpoint	11.25 ± 3.74	10.94 ± 2.97	0.590	0.556	14.59 ± 2.95	12.39 ± 3.80	4.218	<0.001

## Data Availability

The data in this study are available on request from the corresponding author.
